# Pressure waveform analysis for occlusion assessment significantly reduces contrast medium use in cryoballoon pulmonary vein isolation

**DOI:** 10.1007/s10840-024-01801-2

**Published:** 2024-04-17

**Authors:** Vanessa Sciacca, Thomas Fink, Stephan Winnik, Mustapha El Hamriti, Denise Guckel, Maxim Didenko, Maximilian Mörsdorf, Martin Braun, Moneeb Khalaph, Guram Imnadze, Philipp Sommer, Christian Sohns

**Affiliations:** 1 Clinic for Electrophysiology, Herz- und Diabeteszentrum NRW, Ruhr-Universität Bochum, Med. Fakultät OWL (Universität Bielefeld), Georgstr. 11, 32545 Bad Oeynhausen, Germany; 2 Clinic for Cardiology and Angiology, GZO Spital Wetzikon, Wetzikon, Zürich, Switzerland

**Keywords:** Cryoballoon ablation, Pressure waveform analysis, Pressure-guided occlusion assessment

## Abstract

**Background:**

Pulmonary vein (PV) occlusion is crucial for adequate lesion formation during cryoballoon-guided pulmonary vein isolation (CB-PVI). PV occlusion is usually confirmed by angiographies over the inflated balloon device. The aim of our study was to analyze the safety and efficacy of pressure waveform-based PV occlusion assessment during CB-PVI utilizing a novel fully integrated pressure analysis tool.

**Methods:**

Consecutive patients with symptomatic atrial fibrillation (AF) scheduled for CB-PVI were prospectively enrolled for pressure waveform-based PV occlusion assessment. A patient cohort receiving conventional angiographies served as control group. Patients with common PV ostia were excluded.

**Results:**

The study group consisted of 40 patients (16 females, mean age was 64.5 ± 9.7, 45% persistent AF). The control group consisted of 40 matched patients. All 160 PVs in the study group were successfully isolated without the use of additional venograms confirming PV occlusion. The mean procedure duration was 69 ± 12 min in the study group with a mean fluoroscopy duration of 11.5 ± 4.4 min. The mean contrast medium volume was 22 ± 9 ml in the study group and 36 ± 12 ml in the control group (*p* = 0.0001). Mean procedure duration, mean balloon temperatures, and mean ablation application durations did not differ significantly between the study and the control group. No periprocedural complications occurred.

**Conclusion:**

CB-PVI utilizing a fully integrated pressure waveform analysis tool to assess PV occlusion is feasible and safe and significantly reduces the amount of contrast medium without impact on procedural parameters and freedom from arrhythmia recurrence.

**Graphical Abstract:**

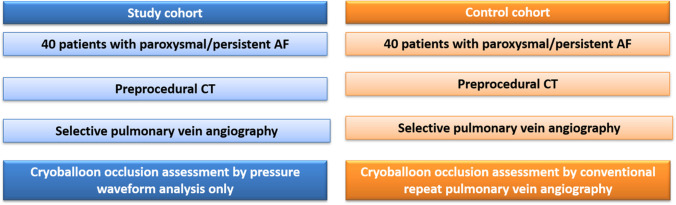

## Introduction

Pulmonary vein isolation (PVI) represents the cornerstone of atrial fibrillation (AF) ablation today [1]. Cryoballoon-guided PVI (CB-PVI) has emerged a well-established ablation approach for safe and successful AF ablation and has been shown to be equally effective to conventional radiofrequency-based ablation approaches [2]. Complete pulmonary vein (PV) occlusion by the inflated cryoballoon is crucial for successful CB-PVI. Therefore, PV occlusion is routinely confirmed by performing contrast medium-based angiographies over the inflated balloon device. However, the risk of renal failure in patients with relevant impaired kidney function, thyroid dysfunction, or allergic reactions is a major limitation of contrast medium utilization. An alternative assessment of adequate PV occlusion during CB-PVI is therefore of great interest for distinct patients at risk of contrast medium-induced complications. A pressure-guided technique to assess ostial PV occlusion has been evaluated in smaller cohorts with varying results [3–5]. A novel pressure waveform analysis tool, which is fully incorporated into the cryoballoon ablation system, enables real-time assessment of PV occlusion during CB-PVI without contrast medium utilization. The aim of this study was to analyze the feasibility, safety, and efficacy of PV occlusion assessment based on pressure waveform analysis during CB-PVI using this novel integrated tool.

## Methods

### Study population

**Fig. 1:**
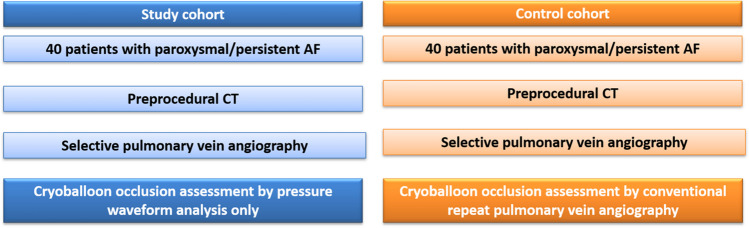
Study flowchart

Consecutive patients with symptomatic paroxysmal or persistent AF scheduled for CB-PVI at our institution were prospectively enrolled for pressure waveform-based PV occlusion assessment (study group). A patient cohort undergoing CB-PVI utilizing PV occlusion assessment by conventional intraprocedural angiographies served as a control group after previous matching of baseline characteristics (control group). The study was approved by the local ethic committee (ethical review board file number 2019–563). All patients gave written informed consent to participate in this study. Figure 1 illustrates the study design.

### Procedural approach

All patients underwent routine preprocedural imaging with computed tomography (CT) to assess the individual PV anatomy. Patients with common PV ostia were excluded from the analysis. CB-PVI was then performed in both groups using the POLARx cryoballoon system (Boston Scientific). The intraprocedural management of CB-PVI has been described in detail before [6–8]. In brief, all procedures were performed under deep analgosedation using propofol, midazolam, and fentanyl. Two right-sided femoral punctures were performed, and a short 8F and 7F sheath was introduced. A diagnostic catheter was placed in the coronary sinus. Single transseptal puncture was then performed using a modified Brockenbrough technique and an 8.5F transseptal sheath (SL1, St. Jude Medical, Inc., St. Paul, MN, USA). After transseptal puncture heparin administration was routinely performed aiming for an activated clotting time ≥ 300 s. Selective angiographies of each PV using a 7F multipurpose catheter or a 7F Amplatz Left 2 catheter were performed in both patient groups. The transseptal sheath was then exchanged over a guidewire for a 15.9F outer diameter steerable sheath (POLAR-SHEATH, Boston Scientific). A 28-mm cryoballoon (POLARx, Boston Scientific) was advanced into the left atrium incorporating a decapolar 20-mm spiral mapping catheter (PolarMap, Boston Scientific) as a guidewire. The cryoballoon was inflated near the PVs and then advanced to the PV ostium aiming at complete occlusion. All PVs were targeted in a clockwise manner beginning with the left superior pulmonary vein (LSPV) followed by the left inferior pulmonary vein (LIPV), right inferior pulmonary vein (RIPV), and the right superior pulmonary vein (RSPV). During right-sided ablation, monitoring of the phrenic nerve was performed by continuous phrenic nerve pacing using a diagnostic catheter introduced into the superior vena cava for tactile examination of the diaphragmatic contraction. Additionally, the diaphragm movement sensor (DMS) was utilized to monitor phrenic nerve activity with a cut-off being set to 60% of diaphragm movement. Ablation was stopped immediately in cases of tactile weakening or loss of diaphragmatic contraction. The standard freeze-cycle duration was set to 180 s, and a time-to-isolation approach was conducted where applicable. In cases where PV isolation was not observed within 90 s of ablation, the freeze was terminated and repeated. Balloon temperatures of − 70 °C or colder led to immediate interruption of ablation. The procedural endpoint was PVI as demonstrated by the circular mapping catheter and stimulation over the diagnostic catheter after the freezing cycle (entrance and exit block). A femoral figure-of-eight suture and a pressure bandage were used for bleeding prevention after sheath removal. Pressure bandages were removed 6 h post-procedure, and sutures were removed the following morning. All patients underwent transthoracic echocardiography immediately following the procedure and the next morning for rule out of pericardial effusion. Oral anticoagulation was re-administered 6 h after ablation and continued for at least 3 months. All patients were treated with proton pump inhibitors for 4 weeks. Anti-arrhythmic drugs were not administered routinely post-ablation.

### Cryoballoon occlusion assessment

**Fig. 2:**
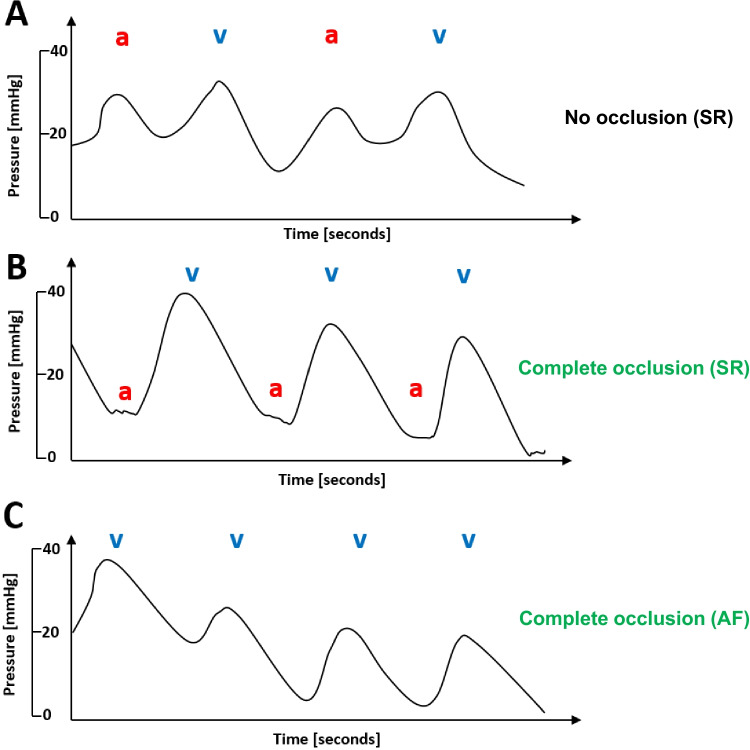
Pressure waveform analysis. **A** A typical pressure waveform in a patient with sinus rhythm before pulmonary vein occlusion is depicted. A and V mark the atrial and ventricular pressure waves. **B** A typical waveform in case of complete pulmonary vein occlusion in a patient in sinus rhythm (SR) is shown, which is characterized by complete loss of the atrial wave. **C** A typical pressure waveform in a patient in atrial fibrillation (AF). No atrial wave can be detected before or after PV occlusion, but a typical increase in the ventricular wave is observed after complete occlusion is achieved

In the study group, PV occlusion was demonstrated only by pressure waveform analysis using a fully integrated tool (SMARTFREEZE Pressure tool), allowing for real-time visualization of pressure curves and values on the screen of the cryoablation console (SMARTFREEZE console, Boston Scientific). The monitoring electric cable of a compatible pressure transducer was connected to an inter-connection box (Boston Scientific) that is linked to the system console. The upper part of the transducer was then connected to a pressurized saline bag, while the lower part was connected to the stopcock of the lateral tube of the hemostatic valve attached to the cryoablation catheter. The pressure transducer was placed on the operation table on the same altitude as the patients’ heart to avoid pressure differences. Pressure waveforms were displayed directly on the cryoballoon console. Interpretation of the pressure waveforms was then performed by the operator. Figure 2 shows examples of typical pressure waveforms obtained during sinus rhythm, where a transition of the pressure waveform from a left atrial to a wedged PV waveform was considered as a complete PV occlusion by the cryoballoon. Pressure waveform analysis in AF was performed analogous. Careful attention was then paid to the detection of typical morphological changes in the ventricular waves by gaining PV occlusion since no atrial waveforms can be seen during AF. In the control group, PV occlusion was demonstrated conventionally by injecting contrast agent over the tip of the inflated balloon device.

### Clinical follow-up

After a blanking period of 3 months, patients completed outpatient clinic visits at 3 and 6 months including standard electrocardiograms (ECG) and 24 h Holter ECG. Telephonic interviews were additionally performed. Recurrence was defined as any episode of documented recurrence of AF or atrial tachycardia for more than 30 s duration outside the 3-month blanking period. Patients with a follow-up duration shorter than 3 months were not included into the analysis. Rhythm follow-up with additional implantable cardiac devices was not performed. Rhythm follow-up was performed analogously in both the study and the control group.

### Periprocedural complications

All periprocedural complications were recorded. Complications were defined as periprocedural when occurring intraprocedural, post-procedural during the hospital stay or until 30 days after the procedure. Complications were classified as major in cases of permanent injury, necessity of interventional treatment, prolonged hospital stay, repeat hospitalization, or death.

### Statistical analysis

Continuous variables were expressed as mean ± standard deviation/standard error of the mean for normal distributions or as median/interquartile range for non-normal distributions. Categorial variables were summarized as counts (%). Comparisons of continuous variables were performed using the Student’s *t*-test for two groups or ANOVAs in the case of multiple groups. Survival analysis was performed using the Kaplan–Meier method. All reported *P* values are two-sided, and a *P* value < 0.05 was considered statistically significant. A propensity score matching was performed with three different matching tolerances (0.2, 0.5, and 1.0) to define the control group. Age, gender, CHA2DS2-VASC score, and type of AF (persistent or paroxysmal AF) were chosen as predictors. A tolerance of 0.5 was accepted for adequate comparability of both sample cohorts, resulting in a 1:1 matching. Estimation of arrhythmia-free survival after CB-PVI was performed using the Kaplan–Meier method for both groups. All analyses were performed using SPPS version 25 (IBM Cooperation, Armonk, NY, USA).

## Results

### Baseline data

The study group consisted of 40 patients (60% males and 40% females). Mean age was 64.5 ± 9.7 years in the study group, and 55% of the patients suffered from paroxysmal AF. The matched control group consisted of 40 patients (62.5% males and 37.5% females) with a mean patient age of 68.3 ± 6.2 years. Paroxysmal AF was present in 60% of the patients in the control group. No patients had implanted cardiac devices within the study or the control group. Table 1 summarizes the baseline data. No statistically significant differences could be observed regarding baseline characteristics between the study groups.

**Table 1:** Baseline data

	Study group	Control group	*P* value
Patients (*n*)	40	40	
Male gender, *n* (%)	24 (60)	25 (62.5)	0.82
Female gender, *n* (%)	16 (40)	15 (37.5)	0.82
Age [years]	64.5 ± 9.7	68.3 ± 6.2	0.21
BMI [kg/m^2^]	27 ± 5	29 ± 4	0.23
Paroxysmal AF, *n* (%)	22 (55)	24 (60)	0.65
Persistent AF, *n* (%)	18 (45)	16 (40)	0.65
CHA_2_DS_2_-VASC-score	2	2	1.0
LAVI [ml/kg^2^]	30 ± 7.2	29 ± 5.4	0.48
LV-EF [%]	53 ± 5.5	55 ± 4.2	0.36

*BMI*, body mass index; *AF*, atrial fibrillation; *LAVI*, left atrial volume index; *LV-EF*, left ventricular ejection fraction

### Procedural data

**Fig. 3:**
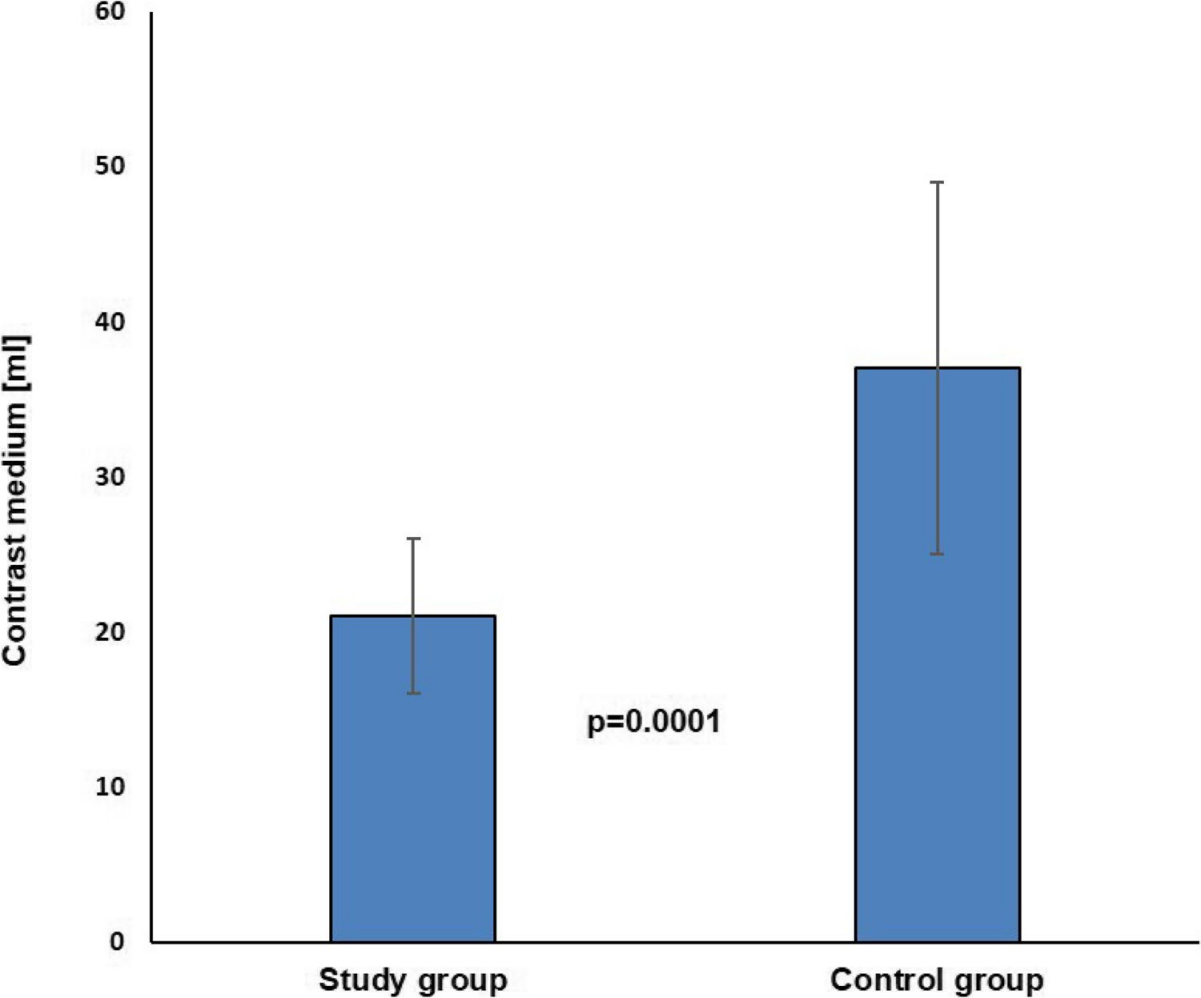
Contrast medium volume. There was a significant reduction in contrast agent in the study group

A total of 320 PVs were targeted in both groups during ablation with 100% of the PVs being successfully isolated by CB-PVI. Mean procedure time was 69 ± 12 min, and mean fluoroscopy time was 11.5 ± 4.4 min in the study group. Mean minimal temperatures during ablation in the study group were − 54 ± 6 °C for the LSPV, − 55 ± 8 °C for the LIPV, − 54 ± 6 °C for the RIPV, and − 51 ± 10 °C for the RSPV. Table 2 summarizes the procedural data for both groups. No statistically significant differences were observed between the two groups in terms of acute procedural success, procedure or fluoroscopy duration, mean minimal temperatures achieved during ablation, mean duration per application, and mean number of applications per vein. No contrast-medium touch-up was necessary in procedures using pressure waveform analysis for PV occlusion assessment. In the study group, the amount of contrast medium used was significantly reduced compared to the control group (22 ± 9 ml study group; 36 ± 12 ml control group; *P* = 0.0001, Fig. 3). In both groups, no major periprocedural complications occurred.

**Table 2:** Procedural data

	Study group	Control group	*P* value
Mean procedure duration [min]	69 ± 12	67 ± 10	0.42
Mean fluoroscopy time [min]	11.5 ± 4.4	10.2 ± 3.8	0.16
Mean minimal temp LSPV [°C]	− 54 ± 6	− 56 ± 10	0.28
Mean minimal temp LIPV [°C]	− 55 ± 8	− 57 ± 4	0.16
Mean minimal temp RSPV [°C]	− 54 ± 6	− 52 ± 7	0.17
Mean minimal temp RIPV [°C]	− 51 ± 10	− 52 ± 9	0.64
Mean number of applications per PV [*n*]	1.4 ± 0.5	1.3 ± 0.7	0.44
Mean duration per application [s]	205 ± 45	196 ± 65	0.47
Contrast medium used [ml]	21 ± 5	37 ± 12	0.0001

*LSPV*, left superior pulmonary vein; *LIPV*, left inferior pulmonary vein; *RSPV*, right superior pulmonary vein; *RIPV*, right inferior pulmonary vein

### Arrhythmia free survival

**Fig. 4:**
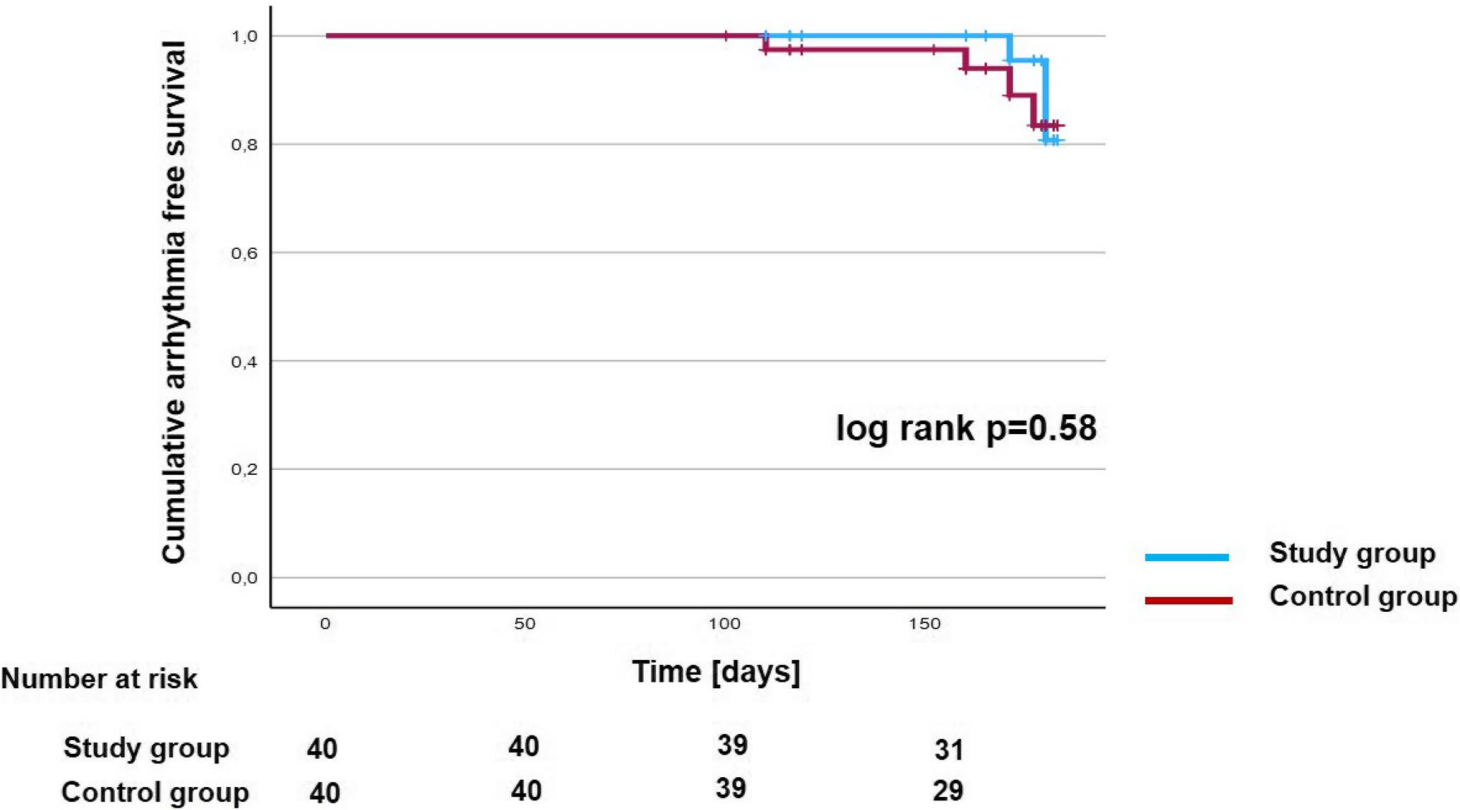
Kaplan–Meier estimation of arrhythmia-free survival. Kaplan–Meier estimation of arrhythmia-free survival after single procedure was 80.8 ± 10.3% in the study group and 83.4 ± 8% in the control group after 6 months (log-rank *P* = 0.58)

Mean follow-up duration in the study group was 141.2 ± 33.5 days and 144.6 ± 31.5 days in the control group (*P* = 0.64). Arrhythmia recurrence after single CB-PVI outside of the blanking period occurred in 3 patients (7.5%) of the study group and in 4 patients (10%) of the control group. At the timepoint of the last follow-up, 37 patients (92.5%) in the study group and 36 patients (90%) in the control group were still in sinus rhythm. Kaplan–Meier estimation of arrhythmia-free survival after single CB-PVI was 80.8 ± 10.3% after 6 months for patients in the study group and 83.4 ± 8% for patients in the control group (Fig. 4). No statistically significant difference in estimated freedom from arrhythmia recurrence was observed between the two groups (log-rank *P* = 0.58).

## Discussion

To the best of our knowledge, this was the first prospective study to evaluate procedural and clinical outcomes of a novel fully integrated pressure waveform analysis tool for the assessment of PV occlusion during CB-PVI. The study has the following major findings.

First, acute PVI could be achieved in all patients undergoing CB-PVI using the pressure waveform analysis tool as the only method to determine PV occlusion. Second, CB-PVI using pressure waveform analysis for PV occlusion assessment was associated with a significant reduction of contrast agent; and third, CB-PVI using pressure waveform analysis had no impact on procedural parameter or patients’ safety.

### Pulmonary vein occlusion assessment by pressure waveform analysis

Siklódy and colleagues initially described pulmonary vein occlusion assessment by pressure waveform analysis during CB-PVI as an alternative concept to conventional contrast agent-based PV angiographies for occlusion assessment over the balloon device [9]. The authors found in a relatively small patient collective of 12 patients that once a PV is occluded by the cryoballoon as demonstrated by simultaneous transesophageal echocardiography, the pressure curve registered at the distal tip of the device inside the vein converts from a left atrial pressure curve to a wedged pulmonary artery pressure waveform. Major limitations of this initial data on pressure waveform analysis to guide CB-PVI were the small patient cohort, the fact that all procedures were performed with a first-generation cryoballoon that is no longer available and that all patients were in sinus rhythm during pressure waveform analysis. Kosmidou and colleagues described PV occlusion assessment by pressure waveform analysis during CB-PVI in a larger patient cohort [10]. The authors found that continuous monitoring of PV occlusion during ablation is an effective approach to assess PV occlusion with accurate prediction of successful PVI. The authors also found a significant decrease in fluoroscopy time and volume of contrast agent when implementing PV pressure waveform analysis. However, all procedures were performed also with an outdated first-generation cryoballoon. In contrast to Siklódy and colleagues, the authors found that pressure waveform analysis for occlusion assessment is likewise possible in patients during AF. Sharma and colleagues were the first to describe the clinical outcome after pressure waveform-based PV occlusion assessment during CB-PVI [11]. The authors found that the single procedure freedom from recurrence of atrial arrhythmia beyond the initial 3 months post-ablation was relatively high with 81.2% at a mean follow-up of 237 days. However, the authors followed a CB-PVI approach using the second-generation cryoballoon including segmental radiofrequency-based ablation, which makes the clinical results less comparable to recent CB-PVI approaches. Furthermore, procedural feasibility in terms of procedure duration has not been systematically assessed.

Raizada and colleagues were the first to give insights on procedural aspects of pressure waveform-based PV occlusion assessment using the second-generation CB [12]. The authors found that pressure-guided CB-PVI significantly reduced the volume of contrast agent as well as fluoroscopy time without compromising acute procedural success in comparison to conventional angiography-based ablation approaches. However, no analysis of long-term arrhythmia freedom was provided in this study. Furthermore, the authors used two 180 s cryoenergy applications for pressure-guided PVI while using two 240 s applications in the angiography-guided group, which may bias the findings on acute success rates. Hasegawa and colleagues were the first to publish a prospective trial on the use of pressure waveform-based CB-PVI with a second-generation cryoballoon [13]. The authors found that pressure-guided CB-PVI was similarly effective and safe compared to conventional CB-PVI with a significant reduction in radiation exposure and contrast agent. The authors excluded patients with a PV anomaly, however, from the study which makes the results applicable only to patients with less complex PV anatomies.

### Pressure waveform analysis by a fully integrated cryoballoon console tool

In contrast to the abovementioned studies on pressure-guided CB-PVI, we present the first prospective data on pressure-guided CB-PVI utilizing the latest generation cryoballoon and utilizing a pressure monitoring tool that is integrated into the cryoballoon ablation system. We could demonstrate that procedural feasibility using this fully integrated pressure analysis tool is high as no procedural prolongation could be noted when performing pressure-based CB-PVI. Furthermore, novel expandable balloon systems may benefit from additional information in terms of PV occlusion when pressure waveforms are analyzed to guide balloon sizing.

### Acute and mid-term success of pressure-based CB-PVI

In contrast to previously published data, we have implemented fixed freezing protocols that were the same in both groups, making our findings on clinical outcomes of pressure-guided CB-PVI more comparable. A major finding in line to the previously published data was that contrast agent could be significantly reduced performing pressure-guided CB-PVI. Notably, a complete contrast-free procedure was not achieved in our setting as we performed PV angiographies in all patients before introduction of the cryoballoon due to the novelty of this fully integrated pressure tool. With a more advanced learning curve, the use of contrast for initial PV visualization may be skipped. Furthermore, the use of intracardiac Doppler echocardiography or PV saline injection under intracardiac echocardiography may further lower the use of contrast. In contrast to previous data, we could not observe significant differences in terms of radiation exposure, which might be an expression of the learning curve in pressure-guided CB-PVI. Importantly, pressure-guided CB-PVI can be used in a complete fluoroless approach when combined with ICE-catheters and 3D mapping as described by Alyesh et al. [14, 15]. Furthermore, we present data on mid-term clinical outcome after pressure-guided CB-PVI. No significant differences could be observed in terms of estimated arrhythmia-free recurrence in both groups at 6 months after ablation. Success rates were comparable to recently published data on CB-PVI [16, 17]. Also, no major complications occurred performing pressure-guided CB-PVI, which accounts for the general safety of this ablation approach.

## Limitations

First, the present study was a single-center study with a relatively small patient population and therefore has the typical limitations regarding data quality and lack of power. Second, patients with PV anomalies as detected by preprocedural imaging were excluded from the analysis. Hence, study findings may be applicable only to patients with less complex PV anatomies. Third, selective angiographies were performed in all patients before ablation. Fourth, the decrease in contrast used was significantly lowered; however, this effect might be more relevant to patients with impaired renal function and less relevant in most patients. Further studies are needed to evaluate the role of pressure waveform analysis-guided PV occlusion assessment aiming for completely contrast-free AF ablation procedures.

## Conclusions

CB-PVI utilizing pressure waveform analysis for PV occlusion assessment is feasible, safe, and significantly reduces the amount of contrast agent compared to conventional angiography-based ablation approaches, without showing impact on procedural parameters. Further studies are needed to address the feasibility of pressure waveform-guided CB-PVI in patients without preprocedural imaging and in more complex left atrial anatomies such as common PV ostia.

## Data Availability

Data will be made available from the authors on reasonable request.
